# Retinal Nerve Fiber Layer Thickness in Prediabetic Patients

**DOI:** 10.5152/eurasianjmed.2022.20420

**Published:** 2022-02-01

**Authors:** Arzu Bilen, Orhan Ates, Osman Ondas, Habib Bilen, Ilyas Capoglu

**Affiliations:** 1 Department of Internal Medicine, University of Ataturk School of Medicine, Erzurum, Turkey; 2 Department of Ophtalmology, University of Ataturk School of Medicine, Erzurum, Turkey; 3 Department of Internal Medicine, Division of Endocrinology and Metabolism, University of Ataturk School of Medicine, Erzurum, Turkey

**Keywords:** Prediabetes, diabetic retinopathy, retinal nerve fiber layer thickness, spectral-domain optical coherence tomography

## Abstract

**Objective:** Diabetic retinopathy is a leading cause of blindness. Diabetic retinopathy is not only seen in diabetic patients with the clinical diagnosis but also in prediabetic patients. The aim of this study is to evaluate the RNFL thickness in prediabetic patients.

**Materials and Methods:** In this study, 50 prediabetic patients and 50 healthy individuals were included. RNFL measurements were performed with SD-OCT in patients with prediabetes and healthy controls.

**Results:** The mean RNFL thickness for the prediabetic group was 94.7 ± 6.3 μm, inferior quadrant (120 ± 11.6), superior quadrant (112.3 ± 14.13), nasal quadrant (71 ± 12.9), and temporal quadrant (65.3 ± 9.2 μm). The mean RNFL thickness for the control group was 98.9 ± 7.5 μm, inferior quadrant (128 ± 14.7), superior quadrant (116.3 ± 15.12), nasal quadrant (77 ± 15.8), and temporal quadrant (71.2 ± 10.3 μm). Variance analysis demonstrated that the RNFL thickness difference between the groups was significant in all quadrants (*P* < .001).

**Conclusion:** RNLF thinning can be seen in prediabetic patients before obvious vascular damage has occurred, and it may present in prediabetic patients not only in the temporal quadrant but also in all quadrants. The early retinal neural changes shown in this study in prediabetic patients may help to better understand the process leading to diabetic overt retinopathy.

## Main Points

- Diabetes mellitus is a major cause of mortality and morbidity along with its microvascular and macrovascular complications.
- Microvascular and macrovascular complications due to diabetes may occur in prediabetics even before diabetes develops.
- In patients with prediabetes, thinning of retinal nerve fiber layer thickness may be present even before diabetes occurs.
- Retinal nerve fiber layer thinning can be seen in prediabetic patients before obvious vascular damage occurs.
- We showed that retinal nerve fiber layer thickness decreased in patients with prediabetes compared to normal individuals.

## Introduction

Diabetes mellitus (DM) is an important cause of morbidity and mortality due to microvascular and macrovascular complications. Diabetes mellitus is an important cause of blindness, end-stage renal disease, neurological complications, lower extremity amputations, and cardiovascular disease. Today, it is known that these complications start from the prediabetes stage, that is, even before diabetes begins.^1^ Diabetic retinopathy (DR) is a leading cause of blindness.^2^ Neurodegenerative and vascular changes may play a role in the pathology of DR.^3^ Neurodegenerative pathologies cause contrast sensitivity, dark adaptation, and electroretinogram abnormalities.^4^ Histological and immunohistochemical studies have shown that neurodegenerative pathologies such as retinal ganglion cells, glial reactivity, glial reactivity lead to a marked reduction in retinal nerve fiber layer (RNFL) thickness.^5^

Diabetic retinopathy is seen not only in diabetic patients with the clinical diagnosis but also in prediabetic patients. Prediabetes refers to conditions when glucose levels and glycosylated hemoglobin values are below the limit for diagnosis of diabetes but above normal limits. In cases of impaired glucose tolerance (IGT), impaired fasting glucose (IFG), and/or A1C in the range of 5.7-6.4%, the diagnosis of prediabetes is considered.^6^ Impaired fasting glucose is defined as 100-125 mg/dL blood glucose level measured after 8-12 hours of fasting. If the blood glucose level measured at the second hour of the oral glucose tolerance test is 140-199 mg/dL, it is considered as IGT.^6^ Prediabetes over time is an important health problem in terms of the transformation of diabetes. Besides, many studies have shown the relationship between cardiovascular disease and prediabetes. Currently, prediabetic patients are being investigated not only in terms of cardiovascular disease but also in terms of microvascular complications. The development of microvascular complications may also occur without obvious diabetes mellitus.^7^

There are no recommendations in the guidelines regarding the presence of possible complications in prediabetic patients and their screening. Fundus examination methods such as direct-indirect ophthalmoscopy, stereoscopy, and digital retinal photography are used to detect retinopathy. Retinal nerve fiber layer thickness measurement is important to determine optic nerve health and nerve injury.^8,9^ Spectral-domain optical coherence tomography (SD-OCT) is a technique that provides a cross-sectional tomographic image of the optic nerve and is used in the early diagnosis of optic neuropathies. This study aims to evaluate the RNFL thickness in prediabetic patients.

## Materials and Methods

This study included 50 prediabetic patients and 50 healthy individuals. Right eyes were evaluated in both groups. Patients were admitted from the ophthalmology department of Ataturk University Training and Research Hospital between January 2012 and July 2016. Consent was obtained from the Ethics Committee of Atatürk University to conduct our study. A comprehensive ophthalmic examination was conducted on participants. Patients with a known ocular disease or a history of surgery were not included in the study. Prediabetes was diagnosed according to the criteria recommended by the American Diabetes Association.^6^ Retinal nerve fiber layer thickness measurements were performed with SD-OCT in patients with prediabetes and healthy controls.

### SD-OCT Scanning Protocols

We used SD-OCT (Optovue, Inc., Fremont, Calif, USA) for the measurement of RNFL in the patient and healthy groups. Retinal nerve fiber layer thickness measurements were performed in all quadrants namely temporal, nasal, superior, inferior, and global.

Windows Statistical Package for the Social Sciences (SPSS) software version 11.5 (SPSS Inc.; Chicago, IL, USA) was used for statistical analysis. A *P* value of < .05 was regarded as statistically significant. All results were expressed as the mean and standard deviation (mean ± SD).

## Results

Fifty prediabetic patients with a mean age of 51.60 ± 5.7 years (range: 32-63 years), 27 (54%) female, and 23 (46%) male were included in the study. Fifty healthy volunteers with a mean age of 50.55 ± 7.1 years (range: 32-64 years), 27 (54%) female, and 23 (46%) males were included in the study as the control group. The age and sex characteristics of the groups were similar.

The mean RNFL thickness for the prediabetic group was 94.7 ± 6.3 µm, 120 ± 11.6 µm for inferior quadrant, 112.3 ± 14.13 µm for superior quadrant, 71 ± 12.9 µm for nasal quadrant, and 65.3 ± 9.2 µm for temporal quadrant. The mean RNFL thickness for the control group was 98.9 ± 7.5 µm, 128 ± 14.7 µm for inferior quadrant, 116.3 ± 15.12 µm for superior quadrant, 77 ± 15.8 µm for nasal quadrant, and 71.2 ± 10.3 µm for temporal quadrant (Table 1).

Variance analysis demonstrated that the RNFL thickness difference between the groups was significant in all quadrants (*P* < .001).

## Discussion

In our study, we showed that RNFL thickness decreased in patients with prediabetes compared to normal individuals. The difference between patients with prediabetes and the control group was significant. The findings of our study support that the SD-OCT method can be used to detect retinal changes that cannot be detected by conventional methods in prediabetic patients before obvious vascular damage occurs.

Diabetes mellitus is a major cause of mortality and morbidity with its microvascular and macrovascular complications. The International Diabetes Federation states that there are 450 million adults with diabetes worldwide.^10^ Half of the diabetics are undiagnosed and do not know that they are diabetic. The prevalence of prediabetes varies between 19.8% and 34.6% due to the different diagnostic criteria of organizations such as the American Diabetes Association and the World Health Organization and is more common than diabetes.^11^ In about 2/3 of prediabetic cases, the metabolic disorder progresses to obvious diabetes in later life.^12^ However, microvascular and macrovascular complications due to diabetes may occur in prediabetics even before diabetes develops. Various studies have shown the development of retinopathy, nephropathy, and neuropathy in prediabetic patients.^13,14^ It is possible to detect early complications in prediabetic patients and to prevent their development. In the Diabetes Prevention Program Outcomes Study, it was shown that intensive lifestyle intervention can prevent the development of diabetes and microvascular complications in prediabetic patients.^14^ This study demonstrated a 21% lower prevalence of retinopathy, nephropathy, and/or neuropathy in women who had been randomized to the intensive lifestyle intervention. The presence of retinopathy in prediabetic patients has been previously shown in some studies. Retinopathy in prediabetic patients has been reported between 8% and 20.9% in these studies.^15^

Retinopathy in diabetic patients is detected by methods such as direct-indirect ophthalmoscopy, stereoscopic photography, and digital retinal photography.^16^ These measurement methods evaluate diabetes-related microvascular damage such as vascular leakage, retinal ischemia, and microaneurysms.^17^ Neuroretinal damage contributes to the pathogenesis of DR and can be seen without symptoms of DR. In our study, we measured the RNFL thickness to determine the neurodegeneration by SD-OCT. This device is widely used in the diagnosis and follow-up of many retinal diseases. Although the effect of DM on microvascular structures is well known, the changes of DM and DR in retinal neurons are not fully known. Even in diabetic patients, changes in retinal neurons may be overlooked by conventional methods.^16^

Neurodegeneration due to loss of ganglion cells in diabetic patients causes RNFL thinning. Studies in diabetic patients have shown that RNFL thinning can be seen before vascular changes have occurred.^5,18^ Also, other studies suggest that RNFL thickness change is associated with oxidative stress seen in the early stages of DR.^19,20^ Although many studies are investigating RNFL by the SD-OCT method in diabetic patients, RNFL examinations in prediabetic patients are very few, and most conventional methods such as fluorescence fundus angiography have been used to show possible retinopathy in prediabetic patients. In human and animal RNFL studies performed by the SD-OCT method in prediabetic patients, RNFL thinning is also reported with or without neurological damage. We have not designed our study to demonstrate the status of the retinal-blood circulation barrier or the relationship between RNFL and various biomarkers that may be an indicator of possible neurological damage. Some studies have been conducted to explain the possible physiopathological mechanisms related to RNFL, but this issue has not been elucidated yet.^21^

Although some studies investigating RNFL thickness by the SD-OCT method in prediabetic patients showed a thinning in the prediabetic, it is reported that the results were not statistically significant.^22^ The difference between patients with prediabetes and the control group was significant in our study. Similar to our study, the Maastricht study is an important study conducted on SD-OCT in 186 prediabetic patients. Retinal nerve fiber layer thickness measurements were performed in all quadrants namely temporal, nasal, superior, and global in this study, and researchers have shown that RNFL thinning occurs in individuals with prediabetes, especially in the temporal sector.^16^ Although RNFL thinning was particularly prominent in the temporal sector in the Maastricht study, RNFL thinning was observed in all sectors in our study. Considering the data of our study, RNFL examinations should not focus on the temporal sector in particular, and all quadrants should be carefully examined.

Data on the relationship between prediabetes duration and RNFL thinning are conflicting in the literature. The diagnosis of prediabetes is often overlooked, so medical data on disease histories are often limited, even if the patients included in the studies were diagnosed with prediabetes. Similarly, in our study, medical information about how long patients had been prediabetic was not sufficient. Therefore, we could not evaluate the relationship between our findings and the duration of prediabetes. Besides, the relationship between prediabetic retinopathy and conditions such as body mass index, cholesterol levels, blood sugar regulation, A1C, and albuminuria, which we did not investigate in our study, was investigated in some studies and no definite relationship could be found.^15^ In prediabetic patients, it is not possible to give an idea about the long-term clinical reflections of early RNFL findings detected in our study and other similar studies.

As a result, in patients with prediabetes, thinning of RNFL thickness may be present before diabetes occurs. Retinal nerve fiber layer thinning can be seen in prediabetic patients even before obvious vascular damage occurs. The early retinal neural changes shown in this study in prediabetic patients may help to better understand the process leading to diabetic overt retinopathy.

## Figures and Tables

**Table 1.** Thickness Measurements of the RNFL in Patients with Prediabetes and Control Subjects

RNFL Thickness Per Sector (µm)	Prediabetes (n = 50)	Control (n = 50)	*P*
Mean RNFL thickness	94.7 ± 6.3	98.9 ± 7.5	<.01
Inferior quadrant	120 ± 11.	128 ± 14.7	<.01
Superior quadrant	112.3 ± 14.13	116.3 ± 15.12	<.01
Nasal quadrant	71 ± 12.	77 ± 15.8	<.01
Temporal quadrant	65.3 ± 9.2	71.2 ± 10.3	<.01
RNFL, retinal nerve fiber layer.			
